# An atlas of mouse CD4^+^ T cell transcriptomes

**DOI:** 10.1186/s13062-015-0045-x

**Published:** 2015-04-03

**Authors:** Michael JT Stubbington, Bidesh Mahata, Valentine Svensson, Andrew Deonarine, Jesper K Nissen, Alexander G Betz, Sarah A Teichmann

**Affiliations:** European Molecular Biology Laboratory, European Bioinformatics Institute (EMBL-EBI), Wellcome Trust Genome Campus, Hinxton, Cambridge CB10 1SD UK; Wellcome Trust Sanger Institute, Wellcome Trust Genome Campus, Hinxton, Cambridge CB10 1SA UK; MRC Laboratory of Molecular Biology, Cambridge, CB2 0QH UK

**Keywords:** T helper cell, CD4^+^ T cell, Treg, RNA-seq, Transcriptomics

## Abstract

**Background:**

CD4^+^ T cells are key regulators of the adaptive immune system and can be divided into T helper (Th) cells and regulatory T (Treg) cells. During an immune response Th cells mature from a naive state into one of several effector subtypes that exhibit distinct functions. The transcriptional mechanisms that underlie the specific functional identity of CD4^+^ T cells are not fully understood.

**Results:**

To assist investigations into the transcriptional identity and regulatory processes of these cells we performed mRNA-sequencing on three murine T helper subtypes (Th1, Th2 and Th17) as well as on splenic Treg cells and induced Treg (iTreg) cells. Our integrated analysis of this dataset revealed the gene expression changes associated with these related but distinct cellular identities. Each cell subtype differentially expresses a wealth of ‘subtype upregulated’ genes, some of which are well known whilst others promise new insights into signalling processes and transcriptional regulation. We show that hundreds of genes are regulated purely by alternative splicing to extend our knowledge of the role of post-transcriptional regulation in cell differentiation.

**Conclusions:**

This CD4^+^ transcriptome atlas provides a valuable resource for the study of CD4^+^ T cell populations. To facilitate its use by others, we have made the data available in an easily accessible online resource at www.th-express.org.

**Reviewers:**

This article was reviewed by Wayne Hancock, Christine Wells and Erik van Nimwegen.

**Electronic supplementary material:**

The online version of this article (doi:10.1186/s13062-015-0045-x) contains supplementary material, which is available to authorized users.

## Background

CD4^+^ T cells are critical orchestrators of the adaptive immune system and can be divided into two main groups, T helper (Th) cells and regulatory T (Treg) cells. Most Th cells exist in a naive state and only differentiate into mature, cytokine-secreting effector cells upon activation of the T cell receptor (TCR) by antigen in the presence of cytokines [1]. Each Th cell subtype exhibits distinct functions reflected by the expression of characteristic sets of cytokines. Several Th effector subtypes have been identified. The best characterised are Th1, Th2 and Th17 cells. The Th1 signature cytokine is IFN-γ), while Th2 cells mainly secrete IL-4, IL-5 and IL-13. Th17 cells produce IL- 17A, IL-17 F and IL-22 [1].

Regulatory T (Treg) cells are functionally distinct from the other Th subsets as they suppress and possibly re-direct immune responses. Depending on their *in vivo* provenance they are referred to as thymus-derived tTreg cells or peripherally-derived pTreg cells [2]. The former commit to the Treg lineage during development in the thymus, whereas the latter differentiate from naive CD4^+^ T cells in the periphery [3].

The Th differentiation process is orchestrated by transcription factors (TFs). The first layer of transcriptional regulation is provided by STAT family factors [4] whilst the maintenance of cell identity appears to be controlled by a second layer of TFs, often referred to as master regulators. Each Th cell subtype is associated with a dominant master regulator whose ectopic expression is sufficient to induce the respective effector cell phenotype. TBX21 (also known as T-bet) is responsible for the Th1 subtype [5], GATA-3 determines the Th2 subtype [6,7], RORγt (encoded by a splice isoform of the *Rorc* gene) drives Th17 differentiation [8], and Foxp3 is responsible for Treg commitment [9,10]. The master regulators collaborate in combination with other lineage-restricted TFs, such as HLX [11], c-MAF [12] and AHR [13,14], which promote Th1, Th2, and Th17/Treg fates respectively. However, these factors alone are not sufficient to drive differentiation towards a specific Th fate.

We sought to create a resource to aid investigation of the transcriptional mechanisms underlying Th cell identity. To this end we profiled the transcriptomes of murine naive, Th1, Th2, Th17, splenic Treg, and *in vitro*-induced Treg (iTreg) cells by RNA-sequencing. We used multiple computational analysis tools to quantify gene and transcript isoform expression levels and to analyse differential expression between CD4^+^ T cell subtypes. We anticipate that the datasets presented here will be a valuable resource for further study and so they were compiled into a publicly available online database, ThExpress.

## Results

### Generation of high quality CD4^+^ transcriptome data sets

**Figure 1:**
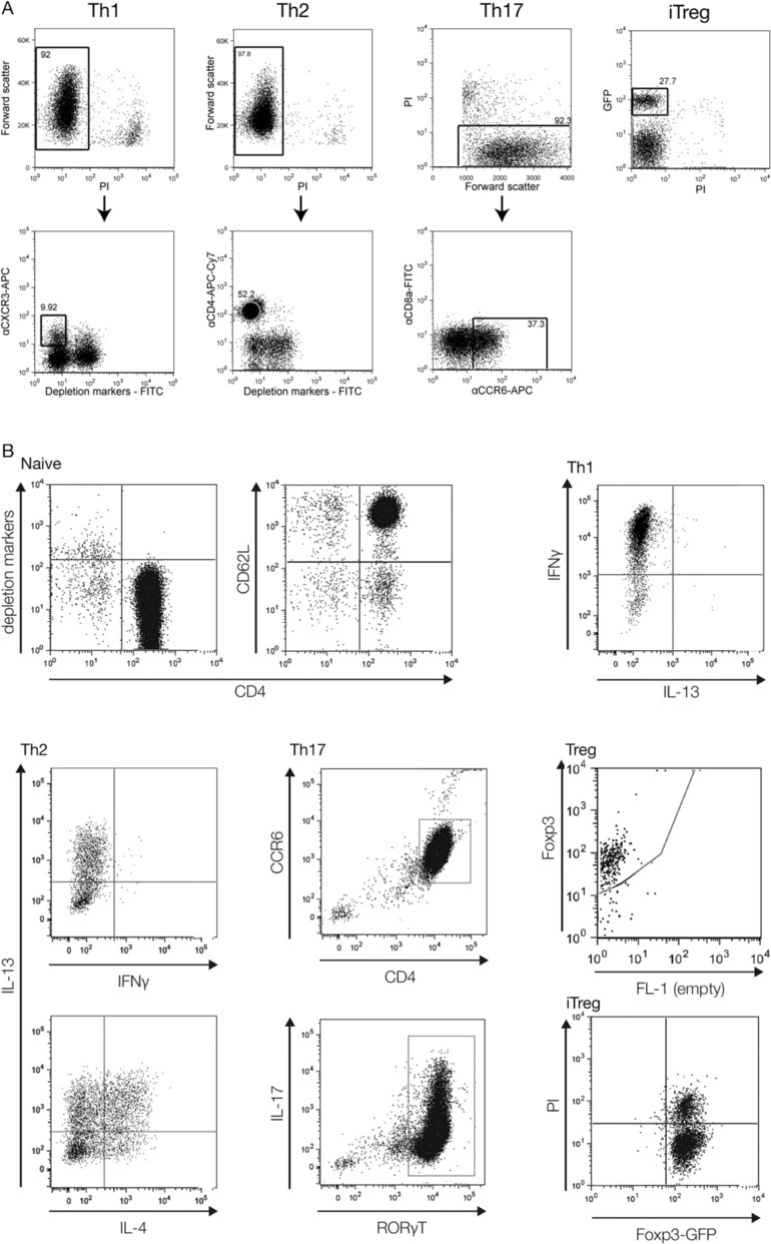
**Flow cytometry sorting and analysis of Th subtype populations. (A)** FACS gating strategies used to sort Th subtypes after growth in polarizing conditions. Initial gates selected for singlet lymphocyte events and were followed by sorting for specific cell surface markers as follows. Th1: CXCR3^+^, PI^−^, depletion markers^−^ (CD11b^−^CD11c^−^Ly6G^−^CD8a^−^CD19^−^). Th2: CD4^+^, PI^−^, depletion marker^−^. Th17: CCR6^+^, Cd8a^−^, PI^−^. iTreg: GFP^+^ PI^−^. **(B)** Verification of CD4^+^ cell lineage identities by intracellular flow cytometry staining for the factors indicated. Cells were analysed using fluorescently-labelled antibodies against the indicated markers. Th1, Th2 and Th17 cells were restimulated prior to analysis as described in Methods. Percentages within the quadrants/gates are indicated, and are representative of the purities routinely obtained.

We performed mRNA-sequencing on two biological replicates of freshly isolated murine splenic CD4^+^CD62L^+^ (naive) T cells and CD4^+^CD25^+^ Treg cells, as well as CD4^+^ cells polarised *in vitro* to Th1, Th2, Th17 and iTreg fates. Lineage identities and differentiation states were verified by analysis of subtype-specific markers (Figure 1). The naive cell samples were over 95% CD4^+^CD62L^+^; Th1 were over 90% IFN-γ^+^IL-13^−^; Th2 were >98% IFN-γ^−^ and 70% IL-4 and/or IL-13 positive. Similar to previous reports [15], we detected significant proportions of cells single-positive for IL-4 and IL-13 under Th2 conditions. Th17 cells were >90% CD4^+^CCR6^+^ and >90% RORγT^+^. Treg purity was confirmed with >90% cells Foxp3^+^. iTreg populations generated from DEREG mice [16] were >95% pure based on expression of transgenic DTR–eGFP under the control of the *Foxp3* locus.

**Figure 2:**
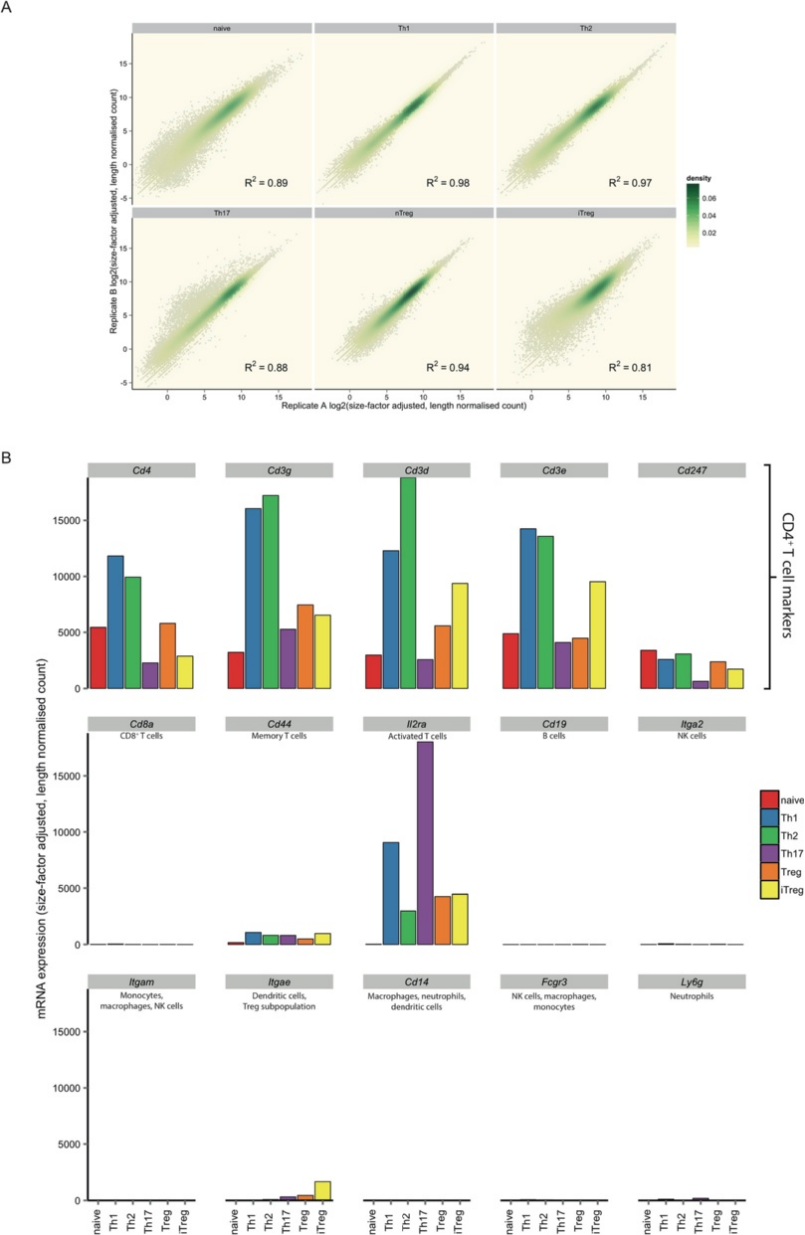
**Quality control of the transcriptomic data. (A)** Comparison of the two biological replicates for each subtype. Gene expression levels as size-factor adjusted, length-normalised counts are plotted for each pair of replicates as two-dimensional kernel density estimates in yellow–green with individual points plotted in light grey. Correlation coefficients are consistently high, ranging from 0.81 to 0.98. **(B)** Expression of splenic cell population markers. Expression levels of markers indicative of CD4^+^ T cells (top row) and those suggestive of other contaminant splenic cell populations (middle and bottom rows).

We obtained between 13.5 and 290 million reads per biological replicate with, on average, 85% mapping unambiguously to the mouse genome (Table 1). We calculated gene expression levels for each sample by normalising raw read counts by size factor [17] and transcript length. Correlations between biological replicates were high (Figure 2).

**Table 1:** **Mapping statistics for the mouse CD4** ^+^ **cell mRNA-seq samples**

**Cell type**	**Total reads**	**Uniquely mapped reads**	**% uniquely mapped**
Naive A	190175282	172714808	90.8
Naive B	291840162	274412494	94.0
Th1 A	101511893	91658389	90.3
Th1 B	86944521	77724382	89.4
Th2 A	102603764	89652943	87.4
Th2 B	103927555	91223060	87.8
Th17 A	83276214	71017085	85.3
Th17 B	289793662	226405227	78.1
iTreg A	13501869	10031784	74.3
iTreg B	53187059	39277954	73.8
Treg A	33430348	28411431	85.0
Treg B	31409616	26002127	82.8

Expression levels of genes associated with other splenic cell populations and therefore suggestive of contamination were low and thus consistent with pure populations of each CD4^+^ T cell subtype (Figure 2).

**Figure 3:**
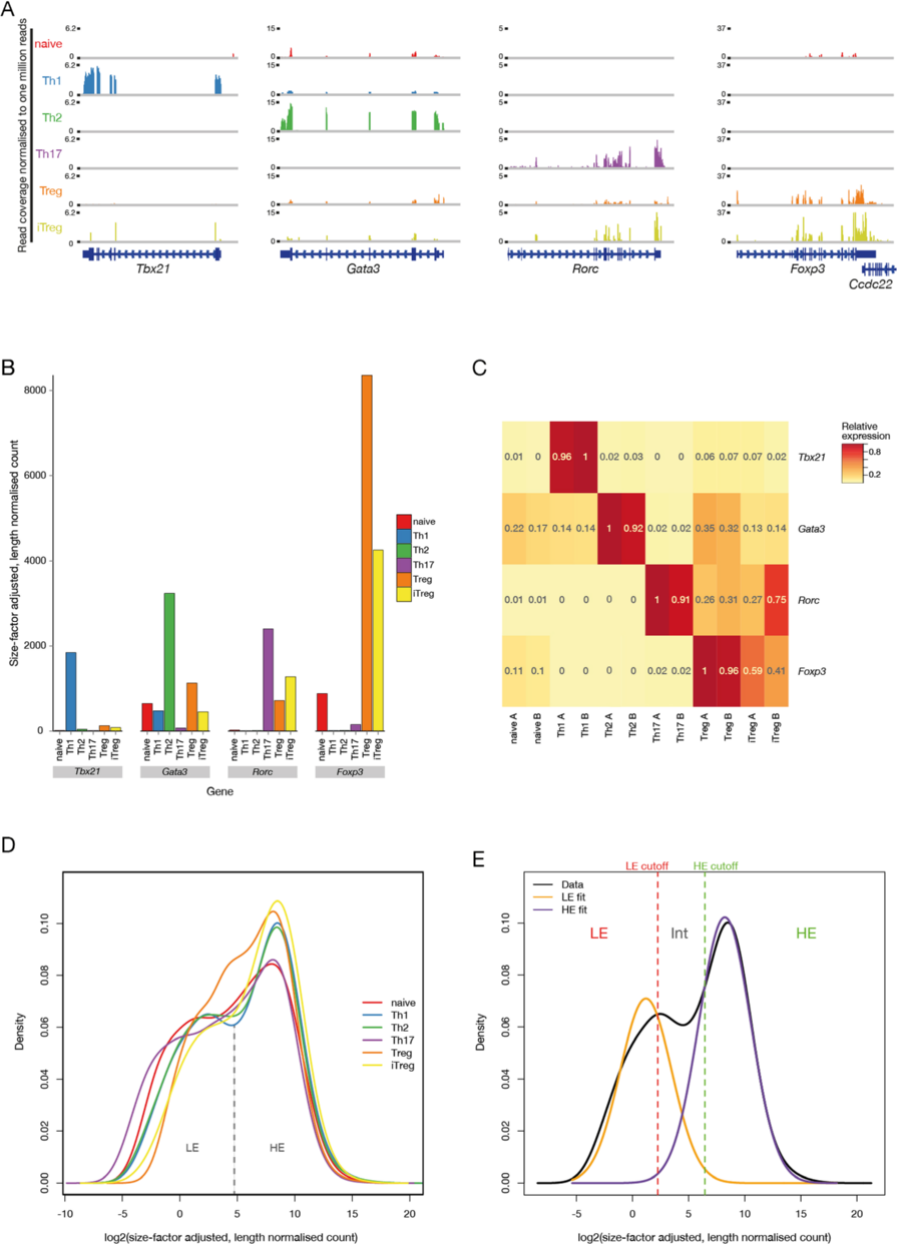
**Master regulator expression and gene expression distributions in CD4** ^**+**^ **subtypes. (A)** Read distributions along the master regulator *Tbx21*, *Gata3*, *Rorc* (RORγt), and *Foxp3* loci in all cell types. The values are normalized to millions of total reads within each library. **(B)** Expression levels of master regulator transcription factors in all cell types. **(C)** Heatmap showing relative expression levels of the transcription factor loci in each biological replicate. Expression levels for each gene are scaled relative to the sample with the highest expression of that gene. **(D)** Kernel density estimates of expression level distributions for all cell types. Genes with zero counts were excluded from the distributions. Each of the samples displays the characteristic low expression (LE)/high expression (HE) shape with a shoulder of lowly expressed genes and a peak of highly expressed. **(E)** Expectation maximisation curve fitting of a Gaussian mixture model for the Th1 expression level kernel density estimate. Kernel density estimate is plotted in black whilst fitted Gaussian distributions are in yellow and purple. Boundaries between LE, Intermediate (Int) and HE expression were calculated using an FDR of 0.01 as previously described [72].

We then used the read distributions at the master regulator transcription factor loci to verify cell type identities (Figure 3A–C). The appropriate lineage markers were expressed at the highest levels in their cognate cell types. In agreement with previously published observations, we did observe low expression levels of some transcription factors within non-canonical cell types: a low level of *Gata3* expression in naive and Th1 cells [7] as well as in Treg and iTreg cell types. GATA-3 is expressed in Treg cells, forms a complex with Foxp3 and is necessary for Treg function [18,19]. mRNA encoding the Th17 regulator RORγt (encoded by a splice variant of *Rorc* which lacks the first two exons) is expressed in the Treg subtypes in agreement with existing work [20]. RORγt interacts with Foxp3 [21,22] and thus might actively contribute to Treg commitment.

### CD4^+^ transcriptomes exhibit two major gene expression classes

We previously demonstrated that genes can be separated into two groups based on their expression level [23] and that this bimodality of gene expression levels is indicative of pure cell populations [24]. The distribution of expression levels in the data analysed here exhibits a peak corresponding to a class of genes with high expression levels (HE), and a shoulder representing genes expressed only at a low level (LE) (Figure 3D–E).

### Transcriptome analysis enables clustering of CD4^+^ cell subtypes

**Figure 4:**
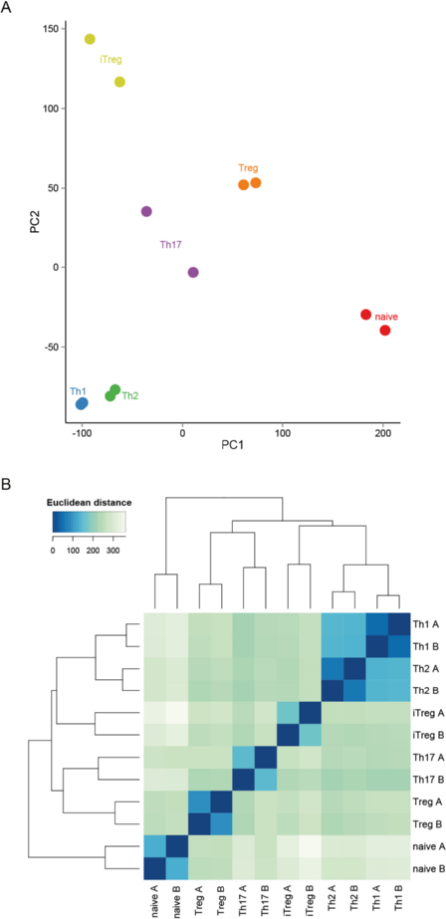
**Clustering CD4** ^**+**^ **subtypes based upon gene expression. (A)** Plot of first two principal components for each sample calculated from regularised-log (rlog) transformed gene expression counts for all genes. **(B)** Heatmap showing hierarchical clustering performed using Euclidean distances for each subtype replicate. Distances were calculated using rlog-transformed gene expression counts for all genes.

To study overall characteristics of the CD4^+^ transcriptomes, we calculated the regularised-log (rlog) transformed expression counts [25] and used these to perform principal component analysis and hierarchical clustering (Figure 4) of the cell types. The individual replicates from each cell type are most similar to each other and the differentiated subtypes cluster separately from naive cells. Furthermore, we found that Th1 cluster with Th2 and Th17 cluster with Treg.

### Clustering of gene expression between subtypes

**Figure 5:**
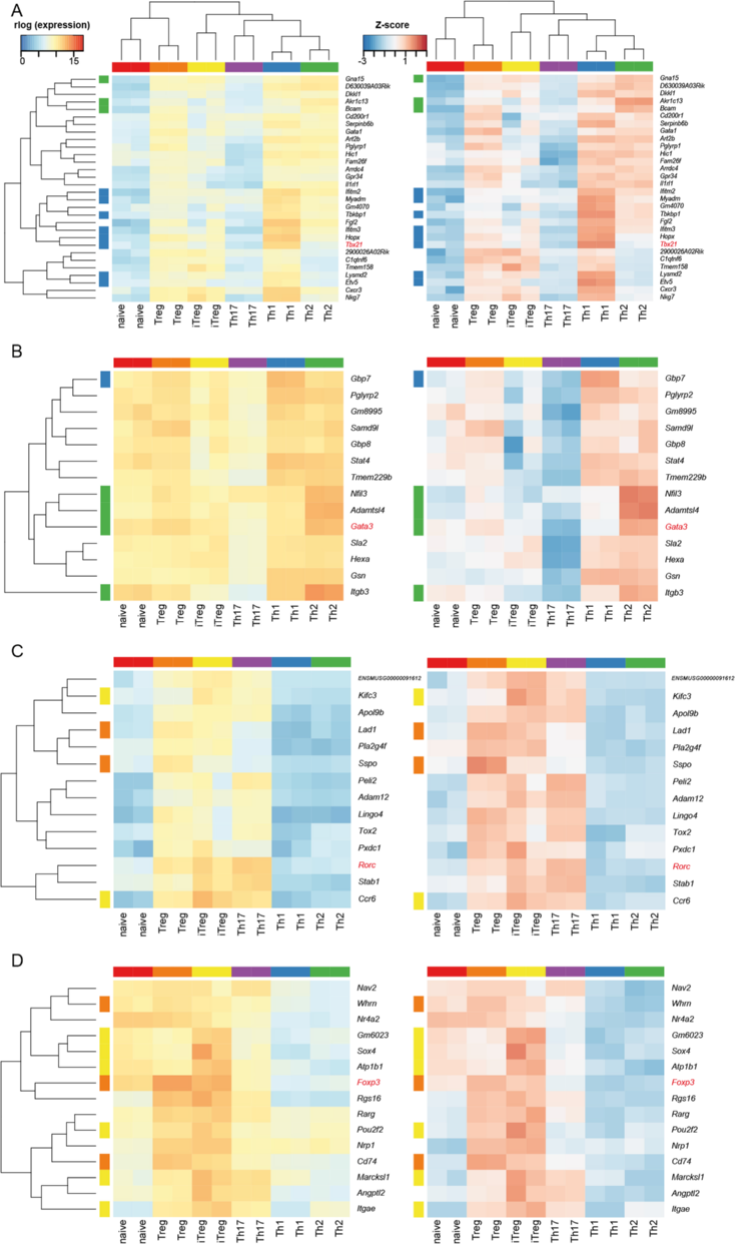
**Clustering of the 50%** **of genes with highest between-sample variance for the CD4** ^**+**^ **subtypes.** Sections of the gene expression heatmap illustrating the local clusters around **(A)** *Tbx21*, **(B)** *Gata3*, **(C)** *Rorc* and **(D)** *Foxp3*. Genes and samples were clustered by Euclidean distance using rlog-transformed expression counts. Expression levels are presented as rlog-transformed counts (left) or as Z-scores (right). Colours to the left of the rows indicate the subtype to which SU genes were assigned.

We used the 50% of genes with the highest variance to perform gene clustering and inspected the clusters associated with known canonical transcription factors to gain assurance that our data will be of use in exploring the regulatory networks involved in CD4^+^ T cell function (Figure 5).

In this analysis, *Tbx21* clusters with *Hopx* and is near to *Fgl2* and *Ifitm3*; *Gata3* clusters with *Nfil3*; *Rorc* clusters with *Ccr6* and *Stab1*; *Foxp3* clusters with *Rgs16* and near to *Sox4* and *Atp1b1*. Reassuringly, all of these associations are expected based on previously published reports of expression or regulation of these genes in CD4^+^ T cell subtypes [26-32].

### Differential gene expression between CD4^+^ subtypes

**Figure 6:**
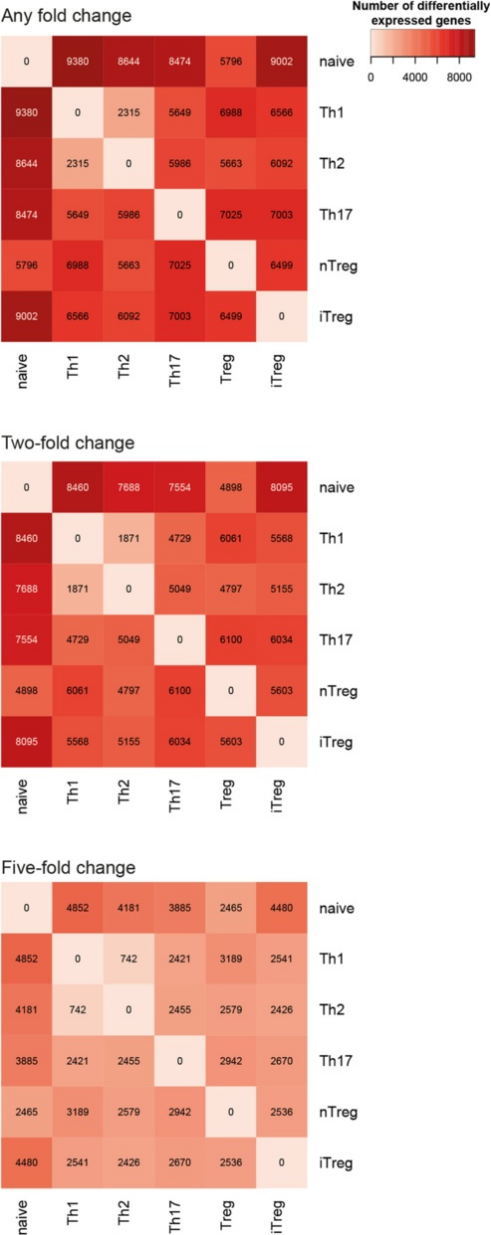
**Differential gene expression analysis between CD4** ^**+**^ **subtypes.** Heatmap representation of the number of genes found to be significantly differentially expressed (p-value ≤ 0.01) in each pairwise comparison at fold change cutoffs of zero, two and five. Lighter colours indicate greater similarity between samples.

A detailed understanding of differential gene expression promises to provide important clues regarding the lineage commitment and biology of the CD4^+^ subtypes. Thus, we determined those genes that exhibit significant differences (adjusted p-value ≤ 0.01) in gene expression between subtypes (Figure 6). The numbers of genes identified as differentially expressed at a significant level range from two thousand (Th1 vs Th2) to over nine thousand.

**Figure 7:**
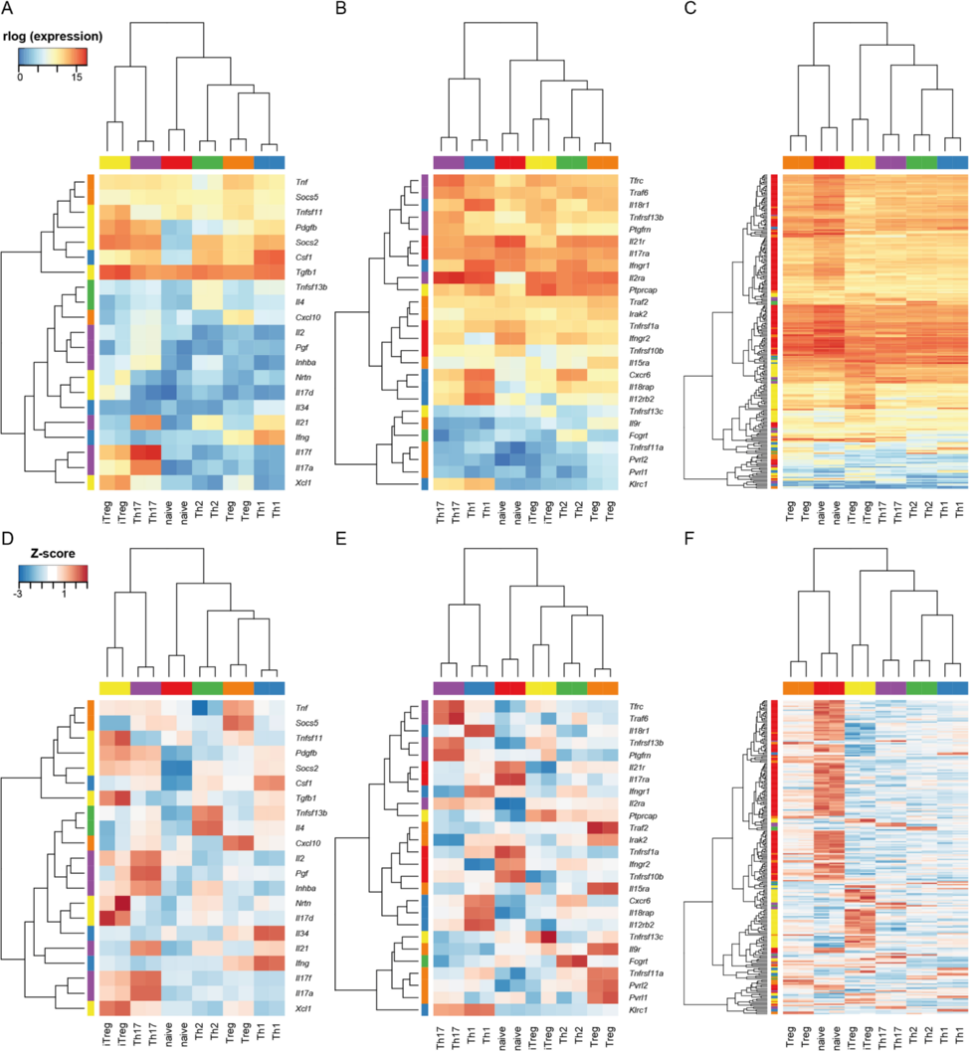
**Expression of subtype upregulated genes for the CD4** ^**+**^ **subtypes.** Expression of SU cytokines **(A, D)**, receptors **(B, E)** and transcription factors **(C, F)**. Expression levels are presented as rlog-transformed counts **(A–C)** or as Z-scores **(D–E)**. Colours to the left of the rows indicate the subtype to which SU genes were assigned.

**Figure 8:**
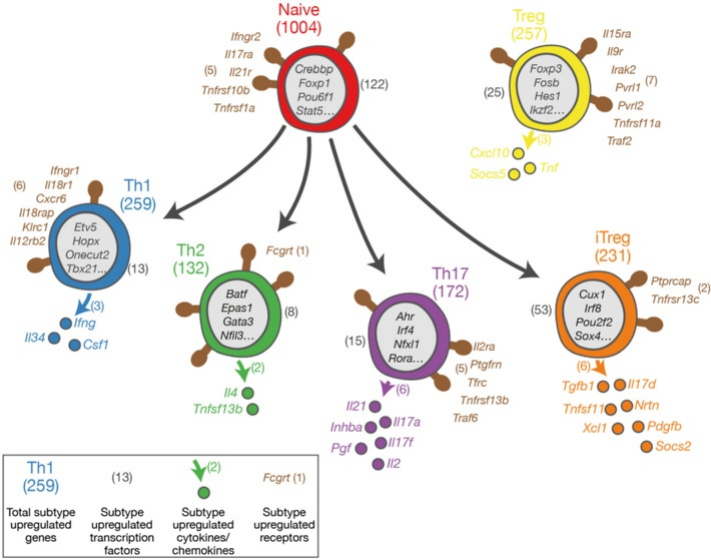
**Scheme illustrating subtype upregulated genes predicted from the mRNA-seq data for each cell type.** Transcription factor genes are listed in grey in the ‘nucleus’ of each cell, those for receptors are in brown next to the ‘lollipop’ shapes that represent them, and cytokines are the coloured spheres outside each cell. Not all genes known to have roles in the function of a particular cell type are identified as SU genes. Due to their expression at high levels in more than one cell type. For example, this is the case for *Rorc* which is the master regulator for Th17 differentiation but is also expressed in iTreg cells.

To exemplify the use of the expression atlas we identified sets of ‘subtype upregulated’ (SU) genes for each cell type by determining those genes significantly upregulated in one subtype with respect to all others (Figures 7 and 8). We found between 132 (Th2) and 1004 (naive) SU genes for each subtype. A complete listing of all SU genes in each cell type is presented in Additional file 1.

When interpreting the lists of SU genes it is important to remember that not all genes known to have roles in the function of a particular cell type will be identified as such due to their expression at high levels in more than one cell type. For example, this is the case for *Rorc* (RORγt, Figure 1) which is the master regulator for Th17 differentiation but is also expressed in iTreg cells [20]. In addition, not all subtype-specific cytokines are classified as SU genes in the Th subtypes that were rested after activation and prior to transcriptome analysis; this is true for IL-13 in Th2 cells.

We generated lists of genes likely to encode transcription factors, cytokines and transmembrane receptors (Additional file 1) and determined which of these genes were found within the sets of SU genes for the CD4^+^ subtypes (Figures 7 and 8).

The presence of the already known lineage markers validates our findings, whilst novel SU genes will provide areas for future study. As an exhaustive analysis would be beyond the scope of this study, we provide examples of expected and novel SU genes below.

#### Naive

**Figure 9:**
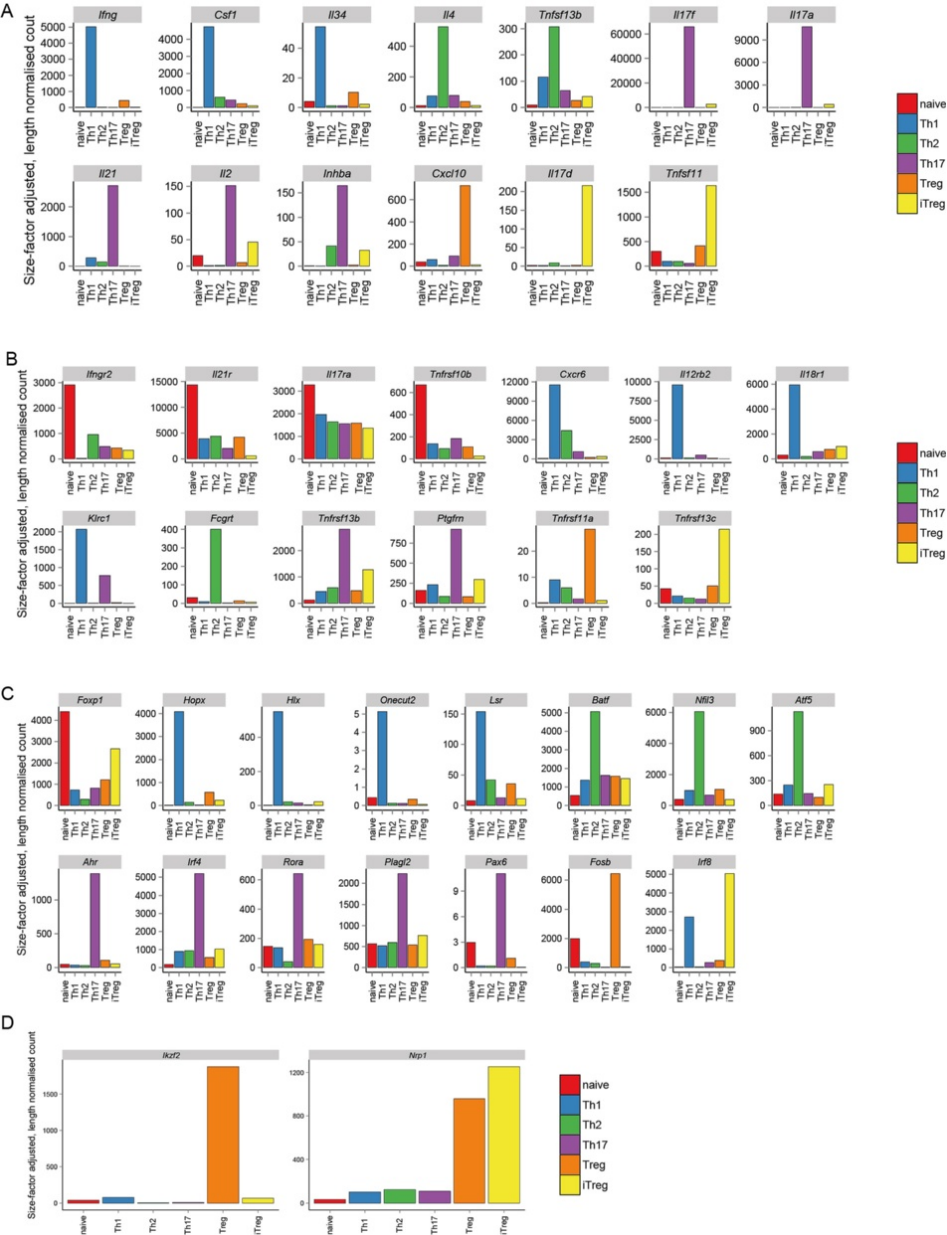
**Expression of specific genes referred to in the text.** Expression levels quantified as size-factor adjusted, length-normalised counts. **(A)** Cytokine genes. **(B)** Receptor genes. **(C)** Transcription factor genes. **(D)** Proposed iTreg/tTreg markers.

As expected, naive cells do not have cytokines or chemokine SU genes (Figures 7,8,9). They do have upregulated receptors *Ifngr2* and *Il21r* conferring the ability to respond to IFN-γ and IL-21 during differentiation to Th1 and Th17 fates respectively. SU receptors previously unknown to play a role in naive CD4^+^ T cells include IL-17RA and the TRAIL receptor (*Tnfrsf10b*). Of the 237 SU genes predicted to encode transcription factors (Figure 7), the majority (123 genes) are associated with the naive subtype. These include *Foxp1* known to be required for naive cell quiescence [33] and STAT5, which is necessary for naive T cell survival [34].

#### Th1

The canonical Th1 cytokine IFN-γ is an SU gene in the Th1 subtype (Figures 7,8,9). CSF-1 and IL-34 are also Th1 SU genes and both act as ligands for the colony stimulating factor 1 receptor (CSF-1R). Th1-associated chemokine and cytokine receptor genes *Cxcr6, Il12rb2* and *Il18r1* [35,36] are upregulated in Th1 cells. An example of a novel SU receptor gene is *Klrc1* which encodes a receptor previously described as having function in NK cells [37].

The gene encoding the canonical transcription factor TBX21 is a Th1 SU gene along with *Hopx, Hlx* and *Onecut2* which all encode transcription factors with known roles in Th1 gene expression [11,26,38]. A potentially interesting novel upregulated Th1 TF is the Lipolysis Stimulated Lipoprotein Receptor (encoded by *Lsr*) which is in the HE class in Th1 cells but in the INT class in all other subtypes.

#### Th2

The well-studied Th2 cytokine IL-4 is a Th2 SU gene (Figures 7,8,9) along with the only other Th2 SU cytokine, B cell activating factor (BAFF, *Tnfsf13b*). There is only a single Th2 SU receptor gene, *Fcgrt*, which encodes a gene with no known function in Th2 biology. Examples of Th2 SU TF genes with known function include *Gata3*, *Batf* and *Nfil3* [27,39]. *Atf5* is a Th2 SU TF gene that has not been characterised in this cell type but has well understood functions in brain [40,41] and liver [42].

#### Th17

Expected Th17 SU cytokine genes include *Il17f, Il17a* and *Il21* (Figures 7,8,9)*.* Whilst IL-2 has been shown to be secreted by Th17 cells [43], its observed upregulation in Th17 cells is most likely due to an experimental bias: the Th1 cells were rested prior to their transcriptomic analysis whilst the Th17 cells were not. Similarly, the IL-2Rα chain (CD25) is particularly highly-expressed in the Th17 cells and so is classified as an SU gene for this subtype; *Il2ra* is highly expressed on all subtypes apart from naive. Of the other Th17 SU receptor genes (including *Tnfrsf13b* and *Ptgfrn*), none have a known T cell function. The TGF-β family cytokine Inhibin A (*Inhba*) is an Th17 SU gene although its secretion by Th cells has not previously been observed.

The transcription factors AHR, IRF4 and RORα are all known to be involved in defining the Th17 transcriptional program [13,20,44] and are defined as Th17 SU genes here. Several novel TF genes were identified, amongst them *Plagl2* and *Pax6*. The former has no known function in T cells, but is a proto-oncogene with roles in acute myeloid leukaemia [45]. PAX6 is well known for its role in brain and eye development [46] and, whilst it is not expressed at the same very high level in Th17 cells as the canonical TF *Rorc*, its expression level is sufficiently high to be of interest.

Th17 cells also express high levels of *Cyp11a1*, a gene for a key enzyme in steroid hormone biosynthesis (data not shown) that we recently found to have a functional role in a subset of Th2 cells [47]. Furthermore, these data confirm our observation that genes of the steroid biosynthesis pathway are upregulated in Th1 and Th2 subtypes and extend this finding to show that they are also upregulated in the other subtypes compared with naive cells.

#### Treg and iTreg

SU gene analysis of the Treg and iTreg populations provides genes that may be useful in distiguishing these two subtypes. Currently, neuropilin-1 (*Nrp1*) and Helios (*Ikzf2*) have been described as markers specific to tTreg cells [48]. In the datasets described here, we see Helios only expressed in the splenic Treg population whilst *Nrp1* is expressed in Treg and iTreg populations (Figure 9). Cytokine genes specific to splenic Tregs include *Cxcl10* whilst *Il17d* and *Tnfsf11* (RANK ligand) are specific to iTregs (Figures 7,8,9). *Tnfrsf11a* (RANK) is an SU receptor gene for Tregs and BAFF receptor (*Tnfrsf13c*) is an upregulated receptor in iTregs. *Fosb* and *Irf8* are transcription factors that differentiate iTregs from Tregs.

### Differential isoform expression

**Figure 10:**
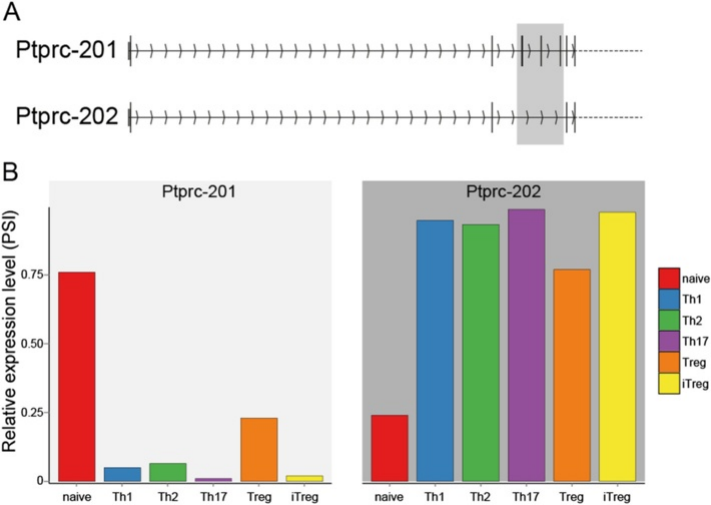
**Alternative splicing of the gene encoding CD45 (** ***Ptprc*** **). (A)** The first exons of the two *Ptprc* isoforms (Ptprc-201, Ptprc-202) annotated in the ENSEMBL GRCm38v70 mouse genome. Exons 4–6 (highlighted by grey box) are skipped in Ptprc-202 leading to a shorter transcript that is preferentially expressed following T cell activation. **(B)** Relative expression of Ptprc splice isoforms in the CD4^+^ subtypes was estimated using the mixture-of-isoforms (MISO) model to calculate percent spliced in (PSI) values for each isoform in each subtype.

Alternative splicing plays an important role in the regulation of eukaryotic gene regulation. To demonstrate the use of our data beyond gene-level differential expression we estimated isoform expression profiles in the subtypes using the mixture-of-isoforms (MISO) model [49]. We observed the previously reported change in major isoform for CD45 (*Ptprc*) [50] (Figure 10) and explored other aspects of alternative splicing during CD4^+^ T cell differentiation.

**Figure 11:**
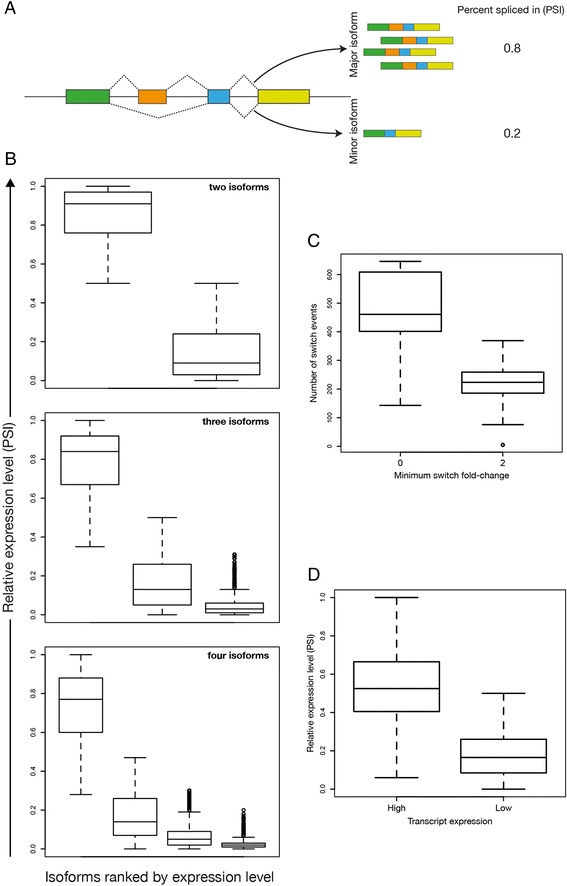
**Differential splicing between CD4** ^**+**^ **subtypes. (A)** Schematic illustrating expression of major and minor mRNA transcript isoforms by alternative splicing of a single gene. **(B)** Distribution of relative expression (MISO percent spliced in, PSI, values) of major and minor isoforms for protein coding genes in Th1 cells with two (top), three (middle), and four (bottom) annotated isoforms. The data represent the top 25% most highly expressed genes with multiple isoforms, such that most of these isoforms are classified as highly expressed (HE) genes. **(C)** For each pairwise comparison between subtypes, protein-coding genes that do not exhibit differential gene expression but that do express different major splice isoforms (referred to as transcript switching) were found. Distributions of the number of genes that exhibit any transcript switching (left) or at least a two-fold switch (right) for all pairwise comparisons between CD4^+^ subtypes are shown. **(D)** Relative expression (PSI) of switched transcripts at their high levels (left) and low levels (right) for each switch event across all comparisons.

For protein-coding genes with multiple isoforms we typically observed a major isoform that comprised the majority of the gene’s total expression while any further isoforms contributed decreasing proportions (Figure 11 A and B). This is similar to the distribution of isoforms previously described in human cells [51].

The major mRNA transcript from a gene can vary between cell types without there being overall differential expression of the gene [51]. Across all pairwise comparisons between cell subtypes, on average, 475 genes exhibited this transcript-switching behaviour whilst 212 underwent at least a two-fold switch (Figure 11C). The expression levels of switched transcripts before and after switching are similar to those of major and minor transcripts from the whole population (Figure 11D).

**Figure 12:**
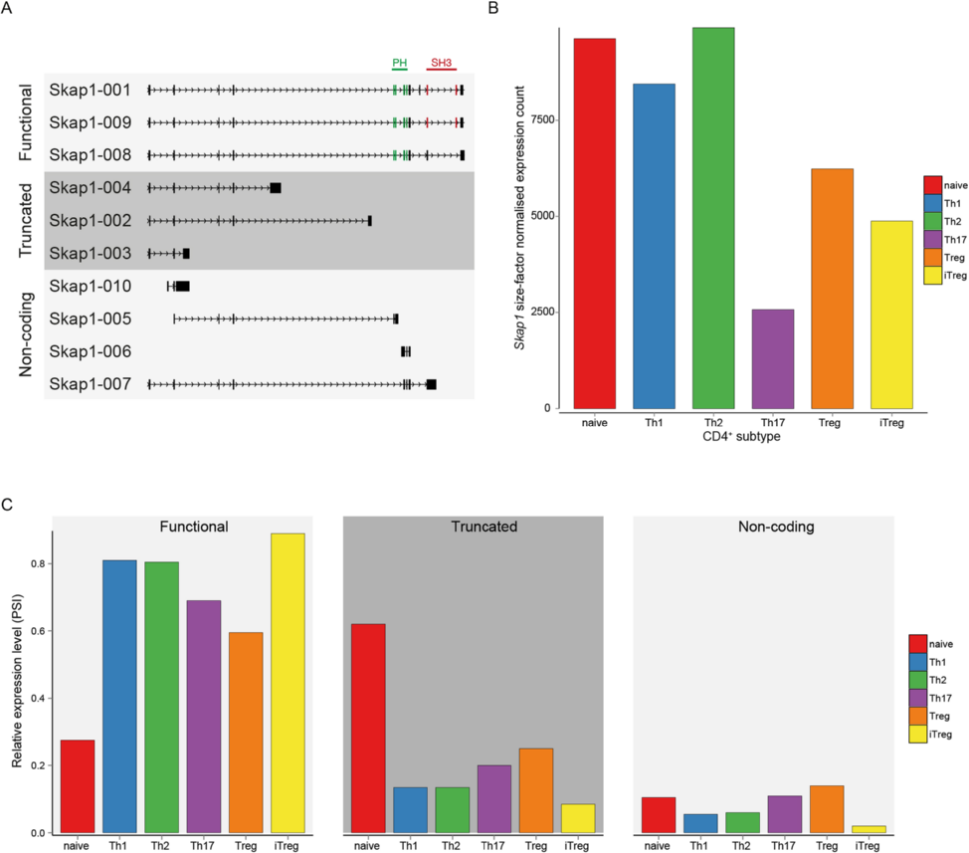
***Skap1*** **transcript switiching. (A)** Splice isoforms of *Skap1* annotated in the GRCm38v70 genome. The ‘functional’ isoforms encode a protein with a PH domain annotated in Pfam; isoforms 001 and 009 also contain an SH3 domain. Exons contributing to the domains are indicated in green (PH) and red (SH3). The truncated isoforms lack both PH and SH3 domains. Non-coding isoforms do not encode a protein. **(B)** Expression levels of *Skap1* in the CD4^+^ subtypes. **(C)** Relative expression of *Skap1* functional, truncated and non-coding isoforms for each CD4^+^ subtype. PSI values were summed for all full-length, truncated or non-coding isoforms.

Isoform quantitation can also provide new insights into gene regulation: the gene encoding the signalling adaptor SKAP-55 (*Skap1*) is an example. SKAP-55 is crucial for integrin activation following T lymphocyte stimulation and contains an SH3 domain and a pleckstrin homology (PH) domain [52-54] required for membrane localisation and protection from degradation respectively [52,55,56]. *Skap1* has ten different splice isoforms (Figure 12A) and, whilst *Skap1* is expressed at high levels in all subtypes (Figure 12B), there are striking differences in its isoform expression. Naive cells predominantly express the shortest truncated form lacking both SH3 and PH domains whilst in all differentiated subtypes the major *Skap1* isoform encodes full-length protein (Figure 12C).

**Figure 13:**
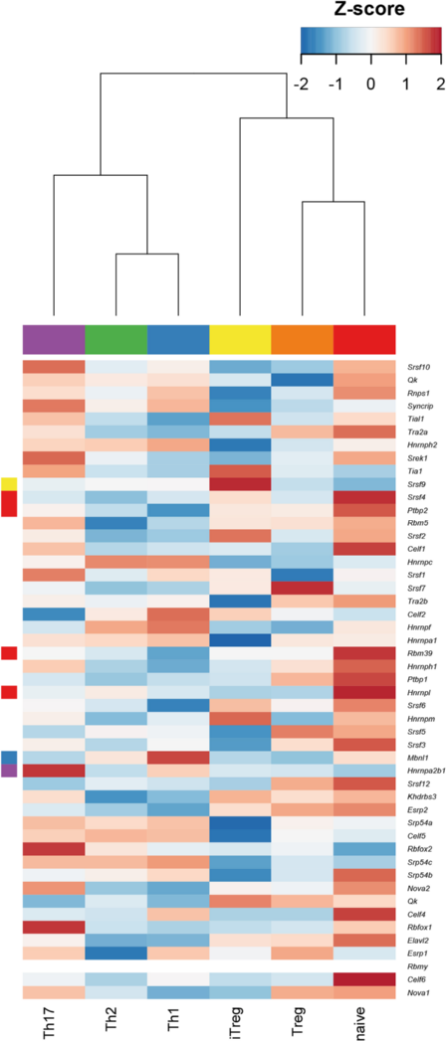
**Varying expression of splicing factors in the CD4** ^**+**^ **subtypes.** Expression levels of genes known to encode proteins involved in pre-mRNA splicing are presented as Z-scores. Colours to the left of the rows indicate the subtype that SU genes were assigned to as follows. Naive, red; Th1, blue; Th2, green, Th17, purple, Treg, orange; iTreg, yellow. The list of splicing factors was taken from Chen et al. [68] and mouse orthologues of human genes were found by searching the Mouse Genome Database [82].

To find a possible explanation for the observed splicing changes between subtypes we analysed the differential expression of genes encoding protein factors involved in the regulation of alternative splicing [57]. The expression of these genes varies between subtypes and seven of them are classed as SU genes (Figure 13).

## Discussion

In accordance with our previous observation in Th2 cells [23], we demonstrated that gene expression profiles from the mRNA-seq data exhibited bimodality with high and low expression classes. This suggests that this expression pattern is a general phenomenon. Several lines of evidence indicate that the HE genes represent the functional transcriptome, while LE genes correspond to transcriptional background [23], enabling us to use HE/LE categories in our analyses of the transcriptomes. The bimodality of expression levels is indicative of pure cell populations [24] and as such increases our confidence in the purity of our starting populations.

Hierarchical clustering demonstrated that naive cells were distinct from all the differentiated subtypes. Th17 and Treg subtypes clustered together which is interesting since both Treg and Th17 gene expression programs can be simultaneously induced in some cells, and some Treg cells appear to be able to transdifferentiate into Th17 cells [20,58]. We also performed hierarchical clustering of the genes with highest between-subtype variability. Genes near to the master regulator transcription factors provided confidence that the coexpression of genes observed in these datasets can be used to investigate CD4^+^ T cell gene regulatory networks.

We determined the subtype upregulated genes that are overexpressed in one cell type compared with all others. Novel SU cytokines, chemokines and cell-surface receptors provide suggestions of previously unknown signalling interactions within the CD4^+^ T cell compartment and also between these cells and other immune effectors. For example, the TRAIL receptor gene (*Tnfrsf10b*) is upregulated in naive cells. Binding of the TRAIL ligand to its receptor leads to apoptosis [59]. It is tempting to speculate that this receptor plays a similar role to Fas [60] in apoptosis during T cell peripheral negative selection.

Th1, Th2 and Th17 SU cytokines imply signalling from these cells to influence the differentiation of other immune cells. The Th17 SU gene inhibin A regulates early T cell development [61]. *Csf1* is upregulated in Th1 cells along with *Il34,* and both proteins encoded by these genes are ligands for the colony stimulating factor 1 receptor (CSF1R) promoting survival, proliferation and differentiation of macrophages [62]. Th2 cells express B cell activation factor (BAFF, *Tnfsf13b*) as an SU gene; BAFF is required for maturation and survival of B lymphocytes. These findings suggest that Th1 and Th2 cells are able to affect the survival and development of the cells with which they interact during an immune response. A receptor for BAFF (BAFFR, encoded by *Tnfrsf13c*) is upregulated in iTreg cells and another receptor, TACI (*Tnfrsf13b*) is expressed on all subtypes raising the possibility that BAFF plays a role in signalling within the CD4^+^ T cell compartment.

If splenic Tregs are similar to tTregs, whilst iTregs are more pTreg-like, then their SU genes will have application in distinguishing between these two cell types *in vivo*. This supposition is supported by the expression of Helios only in splenic Tregs. The proposed tTreg marker *Nrp1* is expressed in both populations in agreement with previous work, showing that it is upregulated during *in vitro* iTreg polarisation [63]. A novel splenic Treg SU gene is *Cxcl10*, a chemokine associated with recruitment of Treg cells to the liver during inflammation [64]. Its expression by Tregs themselves suggests a positive feedback effect whereby Tregs already in the liver are able to recruit more of their subtype. RANK ligand and its receptor, RANK, are iTreg and Treg SU genes respectively. Perhaps this is indicative of signalling between these two subtypes.

We identified 237 SU genes that encoded potential transcription factors, and these will provide a rich resource for investigation into the transcriptional regulation of CD4^+^ T cell fates. Those with notable but low expression within a subtype (such as PAX6 in Th17) may well be a marker for a discrete subset within that population. The highest number (123) of SU transcription factors was found in the naive subtype – perhaps this implies a requirement for this number in maintaining a transcriptional programme poised for commitment to one of several possible differentiated fates. A reduced number of TFs may then be required to maintain a more-specialised fate after differentiation.

Extending comparisons beyond SU genes to find, for example, genes upregulated in two particular subtypes or in all differentiated subtypes compared with naive cells should provide insight into further biologically relevant questions in T cell biology. Examination of gene classes in addition to the TFs, cytokines and receptors described here should also prove fruitful. For example, 49 genes annotated as long non-coding RNAs (lncRNAs) were found to be SU genes.

Estimation of mRNA transcript isoform abundance indicated that hundreds of genes with unaltered between-sample expression levels change their predominant isoform. This demonstrates that regulation of gene function by alternative splicing is a feature of CD4^+^ T cells in addition to erythropoieisis and ESC differentiation [65-67]. We also identified an example of a gene (*Skap1*) that undergoes alternative splicing whereby differentiation away from the naive state leads to increased expression of its full-length, functional isoform. Further work will be required to determine the functional relevance of transcript switching and changes in expression of alternative splicing factors during CD4^+^ T cell differentiation.

As with all transcriptomic studies, the work presented here does not address the question of protein abundance or activity within the subtypes and so conclusions about the role of specific proteins in CD4^+^ T cells should be supported by further work. Furthermore, it should be noted that the transcriptional profiles that we determine here are from single time-points during the development of the subtypes and so do not address the dynamic changes in gene expression that occur during CD4^+^ T cell differentiation; differentiation time-course experiments will provide important avenues for further study. The data presented here do, however, provide multiple insights into the transcriptional landscapes that exist during the maintenance of cell fate once cells have fully differentiated.

## Conclusion

We anticipate that these datasets will be a valuable resource for further study and that our analyses illustrate their utility. We would like these data to be of use to the widest possible range of researchers without the need to use programming environments to perform differential gene expression analysis. As such, we have made the data available in an easily accessible database with a graphical interface and search capabilities at www.th-express.org [68].

## Methods

### Mice and CD4^+^ T cell preparation

C57BL/6 mice were maintained under specific pathogen-free conditions at the MRC Laboratory of Molecular Biology (MRC-LMB) animal facility or at the Wellcome Trust Genome Campus Research Support Facility (both Cambridge, UK). These animal facilities are approved by and registered with the UK Home Office. Animals were sacrificed by approved animal technicians in accordance with Schedule 1 of the Animals (Scientific Procedures) Act 1986. Oversight of the arrangements for Schedule 1 killing was performed by either the Ethical Review Committee of the MRC-LMB or by the Animal Welfare and Ethical Review Body of the Wellcome Trust Genome Campus.

Naive T cells were purified from dissociated spleens from C57BL/6 mice using the CD4^+^ CD62L^+^ T Cell Isolation Kit II (Miltenyi Biotec). Prior to polarisation to the Th17 fate, CD4^+^ CD62L^+^ naive cells were further purified by FACS.

Purified cells were stimulated with anti-CD3e and anti-CD28 and cultured for four days with polarising cytokines and neutralising antibodies. Cells were seeded into anti-CD3 (2 μg/ml for Th1/Th2 or 1 μg/ml for all others, clone 145-2C11, eBioscience) and anti-CD28 (5 μg/ml, clone 37.51, eBioscience) coated 96-well plates, at a density of 250,000–500,000 cells/ml and cultured in a total volume of 200 μl in the presence of cytokines and/or antibodies as follows. Th1: recombinant murine IL-12 (10 ng/ml, R&D Systems) and neutralising anti-IL4 (10 μg/ml, clone 11B11, eBioscience); Th2: recombinant murine IL-4 (10 ng/ml, R&D Systems) and neutralizing anti-IFN-γ (10 μg/ml, clone XMG1.2, eBioscience); Th17: recombinant human IL-6 (30 ng/ml, Immunotools), recombinant human TGF-β1 (5 ng/ml, Sigma), recombinant murine IL-1β (10 ng/ml, Immunotools), recombinant murine IL-23 (10 ng/ml, R&D systems), neutralizing anti-IL4 (5 μg/ml, clone 11B11, eBioscience), neutralizing anti-IFN-γ (5 μg/ml, clone XMG1.2, eBioscience), and neutralizing anti-IL2 (5 μg/ml, clone JES6-5H4, eBioscience). Th17 cells were then FACS sorted for the CCR6^+^CD8a^−^ Propidium Iodide (PI)^−^ population. Th1 and Th2 cells were cultured for another three days without anti-CD3/CD28. The original medium was kept and fresh medium containing the same cytokines as before was added to dilute the cultures 1:3. FACS sorting was used to purify CXCR3^+^CD11b^−^CD11c^−^Ly6G^−^CD8a^−^CD19^−^PI^−^ cells for Th1 and CD4^+^CD11b^−^CD11c^−^Ly6G^−^CD8a^−^CD19^−^PI^−^.

### Treg cell preparation

To generate iTreg cells, CD4^+^ CD62L^+^ cells from DEREG [16] mice were cultured for four days in the presence of anti-CD3e, anti-CD28 as above with the addition of recombinant human TGF-β1 (20 ng/ml, Sigma). GFP^+^ PI- cells were purified by FACS. Splenic Tregs were isolated using MACS by first depleting CD8a, CD11b, CD11c, CD19 and Ly6G expressing cells, followed by positive selection for CD25^+^ cells.

### Flow cytometry analysis

Prior to flow cytometry analysis, Th1, Th2 and Th17 cells were restimulated with phorbol dibutyrate and ionomycin (500 ng/ml, Sigma) for 4 h in the presence of Monensin (2 μM, eBioscience) for the last 2 h. Other subtypes were not stimulated. For staining of intracellular markers, cells were washed in PBS and fixed overnight in IC fixation buffer (eBioscience). Staining for intracellular cytokine expression was carried out according to the eBioscience protocol, using Permeabilization Buffer (eBioscience), and appropriate fluorescent-labelled antibodies. Tregs were permeabilized using the Cytofix/Cytoperm kit (BD) prior to staining. Stained cells were analysed on a FACSCalibur (BD Biosciences) flow cytometer using Cellquest Pro and FlowJo software.

### RNA-seq data generation

Poly-(A) + RNA from all samples apart from Th1 and Th2 was purified, reverse transcribed and prepared for sequencing described previously [23]. For Th1 and Th2 samples the TruSeq RNA Sample Prep v2 kit (Illumina) was used according to the manufacturer’s instructions. The data were deposited at ArrayExpress [69] with accession number [ArrayExpress:E-MTAB-2582].

### RNA-seq read mapping and differential expression analysis

Reads were aligned against the GRCm38v70 mouse genome using the spliced-read mapper GSNAP [70] with only uniquely-mapped reads reported. Expression level quantification was performed using the *htseq-count* Python script [71]. Read counts were used as input to the *DESeq2* R package [25].

*DESeq2* was used to calculate ‘size-factors’ for each sample. Size factors allow for comparison between samples sequenced to different depths in much the same way as simply using the total number of sequencing reads. Importantly, they are not as strongly influenced by differential expression of a small number of highly expressed genes [17]. To permit comparison between genes, size-factor normalised counts were then divided by the length of the transcript to give a measure equivalent to the commonly used reads per kilobase million (RPKM).

To assess distribution of gene expression values, expectation-maximization curve fitting of a Gaussian mixture model and classification of genes into HE and LE classes was performed as described previously [23,72]. Genes with zero read counts were not included. Genes that fell between the expression FDR boundaries were classified as ‘intermediate’ (INT).

Differential expression of genes between subtypes was determined using *DESeq2*. This uses Wald tests to calculate the significance of differential expression for each gene in a comparison between two subtypes. The *p-*values from these tests then undergo Benjamini–Hochberg correction for multiple testing. A gene was considered differentially expressed for a pairwise comparison if its adjusted *p*-value was less than or equal to 0.01. To identify ‘subtype upregulated’ (SU) genes for a particular cell subtype, that subtype was compared with every other subtype. We defined SU genes as those that were significantly upregulated in every comparison and that belonged to the INT or HE classes.

### Gene annotations for transcription factors, cytokines and receptors

Mouse genes were annotated by cross-referencing mouse gene symbols against the following functional annotation databases: mouse Gene Ontology annotations [73], DBD [74], TFCat [75], IMMPORT [76], and Immunome [77]. The following prediction algorithms were used to annotate signal sequences and transmembrane regions: Ensembl’s transmembrane TMHMM [78] and SignalP [79] assignments, and transmembrane helix predictions from Philius [80] and SCAMPI [81]. Genes were categorised as being likely transcription factors, cytokines, receptors, and surface molecules using the criteria described in Additional file 1.

### Hierarchical clustering and principal component analysis

Sequencing read counts were made approximately homoscedastic by using the regularised-log (rlog) transformation provided by *DESeq2* [25]. This transforms the read counts into log space while moderating the differences between samples for genes with low counts so that these genes do not dominate the clustering results.

Hierarchical clustering was performed with rlog transformed gene expression values using the *heatmap.2* function from the *gplots* R package, using Euclidean distance as the distance metric.

Principal component analysis was performed using the *prcomp* function in R.

### Differential splicing analysis

Relative expression of each transcript annotated in the ENSEMBL GRCm38v70 mouse genome was calculated using the mixture-of-isoforms (MISO) model [49]. This provides a percent spliced in (PSI) value indicating the proportion of transcripts estimated to be from each isoform for a gene. The major isoform for each gene in a subtype was found by determining the transcript with the highest PSI value in each biological replicate. If the major isoform was not the same in each replicate then that gene was not included in further analyses.

Transcript switching genes were determined by finding all pairwise comparisons between subtypes where the major isoform differed, but where the gene was not significantly differentially expressed. Genes in the NE or LE expression categories in one or both of the compared subtypes were discounted.

Transcript switching fold changes were calculated as described previously [51]. Expression ratios in each subtype were calculated by dividing the PSI value for the most abundant transcript by that for the other transcript. The switching fold change was then defined as the minimum of the two ratios.

## Open peer review

Reviewed by Wayne Hancock, Christine Wells and Erik van Nimwegen. For the full reviews, please go to the Reviewers’ comments section.

## Reviewer’s report 1

### Wayne Hancock, Perelman School of Medicine, University of Pennsylvania, Philadelphia, USA

This is a useful comparison of the transcriptomes of various murine T helper cell subsets. No data on changes with cell activation or corresponding proteome analysis are available, but the existing information is valuable.

**Authors’ response:***We thank Dr. Hancock for this positive assessment.*

Quality of written English: Acceptable

## Reviewer’s report 2

### Christine Wells, Australian Institute for Bioengineering and Nanotechnology, The University of Queensland, Brisbane, Australia

The manuscript by Stubbington and colleagues describes an RNAseq experiment on mouse T-cell subsets. The manuscript is well written, the experiments designed sensibly - well replicated and sequenced to good depth for the analyses provided here - altogether the manuscript provides a useful description of high quality RNAseq data. The data is available in GEO, and also in a local database, th-express, however this local database was not accessible to me at the time of this review.

My main critique of the manuscript is centered on the interpretation of the data.

On page 4, the authors state their motivation for the study as “To better elucidate the transcriptional mechanisms underlying Th cell identity…”. Actually, this is a descriptive paper, albeit a very useful one, but no attempt has been made to identify mechanism, so this is a little too disconnected from the content of the paper. In the same summary paragraph, the authors went on to promise a computational pipeline - but it is not clear what method development has been undertaken in the manuscript.

**Authors’ response:***We agree that this paragraph could have been better phrased. We have altered it as follows to more accurately represent the work within the paper.*

“**We sought to create a resource to aid investigation of the** transcriptional mechanisms underlying Th cell identity. To this end we profiled the transcriptomes of murine naive, Th1, Th2, Th17, splenic Treg, and *in vitro*-induced Treg (iTreg) cells by RNA-sequencing. **We used multiple computational analysis tools** to quantify gene and transcript isoform expression levels and to analyse differential expression between CD4^+^ T cell subtypes. We anticipate that the datasets presented here will be a valuable resource for further study and so they were compiled into a publicly available online database, ThExpress.”

*We also altered the abstract in a similar manner to include the following text:*

“**To assist investigations** into the transcriptional identity and regulatory processes of these cells we performed mRNA-sequencing on three murine T helper subtypes (Th1, Th2 and Th17) as well as on splenic Treg cells and induced Treg (iTreg) cells.”

I’d like to commend the authors on the thoroughness of their descriptions of data quality and sample quality - I found Figures 1,2,3 both useful and sensible in this regard - this adds to the value of the RNAseq data immeasurably, as it provides context often missing from similar studies. Figures 4 and 5 contain a lot of redundant information, which should be summarized more succinctly, and in relation to the analyses conducted in the next stages of the manuscript. The overall conclusions to be drawn from Figures 1,2,3,4,5 are that the material is from lymphocytes that have transcriptional phenotypes consistent with their known roles as T-cell subsets.

**Authors’ response:***We thank Dr. Wells for her comments on our attempts to characterise in detail the cell populations that we studied. Although Figures*4*and*5*do contain some redundant information, we feel that multiple visualizations of these large and complex datasets serve a valuable purpose in clarifying the analyses that were performed with regards to clustering and the differential expression of genes.*

I dislike the use of ‘signature’ as a description of the genes that were differentially expressed in what appears to be a pair-wise comparison across all of the different sample classes. The term suggests that these genes will be a definitive set of genes that are characteristic of one particular T-cell subset. I would expect that a ‘signature’ should reoccur in other datasets and be indicative of the presence of a particular T-cell subset, and the authors point to this in their discussion of the signature genes. However the authors haven’t run any experiments to assess how stably the expression of these genes might be in terms of temporal expression in culture, *in vivo* or in ex vivo isolated cells, or even in the same subsets taken from different mouse strains or under different or continuous stimulation. There are a number of independent T-cell RNAseq experiments available in GEO that could have been used in some form of cross-validation, but this was not attempted. Quite a few of the genes listed in various signatures - Cxcl10 for example - are inducible genes and may be transiently expressed, not as a signature of a t-cell identity, but rather as a signature of an external stimulus.

**Authors’ response:***Given this comment and Dr. Wells’ recommendation below about an ‘edit of nomenclature’ we have changed our terminology to use ‘subtype upregulated’ instead of ‘signature’ throughout the manuscript.*

The observations about the extent of alternate splicing were very much underplayed in this manuscript, but tantalizing, and I would like to have seen a broader description of these events in the different T-cell subclasses. Re the analysis of splicing factors - this was very shallow. These factors work in complexes and I would have found it more helpful to have the analysis focused on the changes to complexes that might help prioritise candidate isoform-determining events.

**Authors’ response:***We agree that our findings about alternative splicing are tantalizing and certainly are worthy of further study. In this context, we intended only to demonstrate the use of our datasets beyond simple gene-level expression quantification and not to add in detail to the extensive body of literature on the mechanisms of alternative splicing. We hope that this work provides an inspiration and a resource for more in-depth analyses of alternative splicing in CD4*^+^*T cells. We have added the following text to the results and discussion sections to clarify our appreciation of the need for further work.*

“**To demonstrate the use of our data beyond gene-level differential expression** we estimated isoform expression profiles in the subtypes using the mixture-of-isoforms (MISO) model [49].”

“Further work will be required to determine the functional relevance of transcript switching and changes in expression of alternative splicing factors during CD4^+^ T cell differentiation.”

Overall the manuscript describes a valuable dataset, which has been carefully collected from well phenotyped cells and has been analyzed with a great deal of consideration to the importance of this phenotyping. The description of the differentially expressed gene lists may be over-interpreted, specifically in regard to potential T-cell expression signatures. My recommendation would be an edit of nomenclature in the absence of a more rigorous testing of any signature on external data.

**Authors’ response:***To avoid overinterpretation we have changed our nomenclature as discussed above.*

Quality of written English: Acceptable

## Reviewer’s report 3

### Erik van Nimwegen, Biozentrum, University of Basel, Switzerland

Report form: This is a nice and very solid looking study concerning the transcriptomes of different types of T cells, and I have no doubt that this will be a very valuable to the community of T cell researchers. Right at the outset, I'd like to note that I know very little about the biology of different T cell types, so that I am unable to make informed comments on the plausibility, novelty, and general interest of the particular biological observations made in this manuscript. My review will focus predominantly on the computational analysis methods used, as this is my area of expertise.

My overall impression is that the computational analysis is very competently performed. There aren't any particularly novel methods introduced as far as I can see, but the analysis seems appropriate and reasonably done. I felt that the identification of ‘signature genes’ that are over-expressed in a particular cell type relative to all other ones, and the further focus on signature genes within particular functional categories of genes (e.g. receptors, cytokines, TFs), was a particularly nice and useful approach. Its relatively simple but helps focus on those genes that are the best candidates for providing biological insights into what is specific about each subtype.

My only ‘issues’ with the manuscript as it is now concern the descriptions of a number of the methods employed, which often seem unnecessarily cryptic and vague. In some cases this leads me to be unable to assess whether the conclusions that the authors present actually follow from the presented data.

This is partly caused by the fact that the resolution of some of the images is really poor. To give an example, in Figure 2A none of the symbols are readable and also the scatter of the grey dots cannot be clearly discerned. In fact, from what I can tell from the figure in its current resolution it appears as if the grey dots fall within narrow vertical stripes in some of the panels, which would be rather strange. Thus, although I have little doubt that the replicates look fine, based on the current Figure 2A I cannot really assess this.

**Authors’ response:***We were surprised to find that the figures were not of sufficient resolution. Having spoken to the editorial team at Biology Direct we have found that this was, for the most part, due to a problem during conversion after manuscript submission. We will ensure that the final figures are optimal. We did, however, discover a problem with the resolution of Figure*2*A that has now been corrected.*

For the gene expression analysis, the authors make use of the DESeq suite of tools, and in the description of the Methods refer to certain technical concepts associated with this tool such as the 'size factor' and 'r log transforms'. Although I appreciate that DESeq is a fairly widely used tool, it would make the manuscript much more self-contained if one or two paragraphs in the Methods could be spend explaining what these things are and how precisely they are defined. Right now a lot of the Methods are described at the level of 'we used this tool to get this, then that tool to do that', and unless one has specific experience with these tools, it is hard to tell precisely what was done.

**Authors’ response:***We have extended and clarified the methods to add detail about the tools used. The following details were added.*

*“DESeq2* was used to calculate ‘size-factors’ for each sample. Size factors allow for comparison between samples sequenced to different depths in much the same way as simply using the total number of sequencing reads. Importantly, they are not as strongly influenced by differential expression of a small number of highly expressed genes [17]. To permit comparison between genes, size-factor normalised counts were then divided by the length of the transcript to give a measure equivalent to the commonly used reads per kilobase million (RPKM).”

“Differential expression of genes between subtypes was determined using *DESeq2*. This uses Wald tests to calculate the significance of differential expression for each gene in a comparison between two subtypes. The *p-*values from these tests then undergo Benjamini–Hochberg correction for multiple testing.”

“Sequencing read counts were made approximately homoscedastic by using the regularised-log (rlog) transformation provided by *DESeq2* [25]. This transforms the read counts into log space while moderating the differences between samples for genes with low counts so that these genes do not dominate the clustering results.”

For a non-expert, the significance of the beginning of the results section, reporting some statistics on the frequencies of occurrence of various sub-type specific markers, is hard to interpret. In particular, I understand that according to the literature, each subtype is characterized by a particular combination of markers. So ideally, each cell would show one of these known combinations of markers and would thus have an unambiguous subtype. But it was hard for me to tell from the reported results what fraction of cells are unambiguous in this way, or whether there are subsets of cells with previously undescribed combinations of markers.

"Expression levels of genes indicative of contamination.... consistent with pure populations of each CD4^+^ T cell subtype (Figure 2)." I did not understand this analysis. I presume that 'genes indicative of contamination' are genes that should not be expressed at all in the subtype under consideration (it would be nice to explicitly state this). But if that's what they are, then any expression of these genes should be inconsistent with pure populations. Figure 2 does seem to show some expression for some of these genes so I do not understand the claim in the sentence cited above. This is just another example where I think the authors could have easily been much more concrete and specific regarding precisely what analysis was done.

**Authors’ response:***These data are intended to provide readers the opportunity to assess the purity of our starting cell populations using the expression of well-characterised combinations of markers. The high percentages that we observe are consistent with populations accepted to be pure in previous work.*

*Regarding the data in Figure*2*the ‘genes indicative of contamination’ are those that we would expect to see expressed at high levels in populations of other cell types typically found in the spleen. For the most part we see very low expression of these genes within our samples. Very low levels of expression may be due to a small amount of contamination (insufficient to dominate overall gene expression within the sample) or may indicate genuine expression of that gene at low levels within the cell population of interest. To attempt to add clarity to this section we have altered the relevant paragraph and also changed the text to read ‘genes****suggestive****of contamination’.*

The authors find that TFs which are know lineage markers always have higher mRNA levels in their cognate subtype than in all other subtypes. This would imply that all lineage marker TFs are regulated transcriptionally (rather than,say, through post-transcriptional or post-translational mechanisms). I find this striking, i.e. in my experience differences in TF activities are often non-transcriptional in mammals, and wonder if the authors might want to command on this.

**Authors’ response:***We agree that TF activity regulation by non-transcriptional mechanisms is not unusual and this is certainly the case for the STAT proteins upstream of the master lineage regulators described in this work. However, in the case of the well-characterised master regulators it is known that their mRNA expression is highest in their cognate cell types and that ectopic expression of the genes within naive CD4*^+^*T cells leads to differentiation to the appropriate subtype* [5-10]*. This suggests that mRNA levels are a suitable proxy for TF activity in these cases. Of course, this does not rule out an additional role for non-transcriptional mechanisms in the modulation of activity of these factors.*

Regarding the Bimodal expression distributions. I know the authors have previously published on this, but it is any issue of which I must frankly say I am a bit skeptical based on my own experiences with analysis of RNA-seq data. In particular, when we analyze RNA-seq data-sets in my lab, we typically cannot robustly identify such bimodal distributions. Also, I have extensively analyized CAGE data, which has the advantage that it quantifies directly the expression of a TSS (i.e. without having the complication of having different transcript lengths) and the distribution of expression levels of TSSs NEVER look bimodal. They almost always look like power laws. Another issue that makes me sceptical is that, depending on precisely how the RNA-seq data is processed, it is quite easy to produce kernel densities that 'look' bimodal, but this bimodality is more an artefact of the data processing than a true underlying biological reality. In particular, RNA-seq data is inherently discrete, i.e. the read count in a given sample for a given gene (or transcript) is either zero, one, two, three, etcetera. To draw a density of (log) gene expression levels, there needs to be a normalization for length, some sort of 'pseudo-count' needs to be added to deal with genes that have zero counts, the numbers need to be log transformed, and then finally the density is typically approximated using some kernel density. Depending on precisely how this is done one can easily produce a 'low expression peak' that simply corresponds to all genes that had zero read counts, but whose 'expression' levels are smeared out in the density because of their differing lengths and the convolution with the kernel. But this low expression peak is then typically not robust to changes in the pseudo-count or normalization procedure.

Notably, when a gene had a read count of zero, this means its total expression was at most on the order of 1/total_reads_in_sample, but it could be arbitrarily smaller (which is really an infinitely wide range of possibilities in log-scale). Due to this, estimating the density of expression levels in log-scale becomes poorly defined in the region that corresponds to very low read-counts. So to make a convincing case that the distribution is truly bimodal on a logarithmic scale, I feel that quite a bit more needs to be done. One could for example make a cumulative distribution F(x) which shows the expected fraction of all genes that has a log-expression level less than x. To estimate F(x) one would calculate for each gene g, the probability p(>x|g) that its expression is larger than x, given the number of reads it has. F(x) would then be estimated as F(x) = sum_g (1-p(>x|g)). One would then like to see that there is a clear inflection in this curve. There might be other, better, ways to show bimodality. I realize that the authors might feel they have already made a sufficient case for bimodality in previous publications but I thought they might want to be aware that some people, such as myself, have not been convinced that this bimodality is real, and this might be an opportunity to make a case to convince people like me.

**Authors’ response:***Thank you for the detailed discussion on this point. Firstly we would note that genes with zero counts were not included in the bimodality analyses and so did not contribute to the LE population within the kernel density plots; we have added text to the manuscript to clarify this point. Whilst we would like to be able to convince skeptics as to the reality of this bimodal distribution it is beyond the scope of this manuscript to do so. Our previously published work on this topic includes a plot showing the third mode that occurs if genes with zero counts are included (FigureS4 in* [23]*); we see similar results with the data here. We have also published validations of the findings by single-molecule RNA-FISH* [23] *and modeling of the distributions that would occur from populations of varying purity* [24]*. As such, we continue to feel that this is a useful method for use in assessing purity of populations (in conjunction with the other more cell type-specific approaches used here) as well as for determining genes to exclude from differential expression analyses in a rational manner.*

Reqarding the signature gene expression. It is one thing for a gene to be higher expressed in the specific subtype than in all others, but this does not necessarily imply that a measurement of the gene's expression level would be sufficient to realiably classify the subtype. It would be nice if the authors could show, or at least discuss, to what extent the subtype can be identified from expression measurements of a signature gene.

**Authors’ response:***Given these comments and those from Dr. Wells above, we have adjusted our nomenclature to change ‘signature genes’ to ‘subtype upregulated’ genes. We hope that this will avoid overinterpretation of the lists as being definitive of a particular subtype. We intend them to act as a demonstration of the utility of our datasets whilst providing possible avenues for further work regarding the functional roles of particular gene products in the various subtypes.*

It appears that the naive subtype has by far the largest number of TFs that are signature genes (123 or 237). Can the authors comment on this observation?

**Authors’ response:***This is an intriguing finding. We added the following text to the discussion to address it.*

“The highest number (123) of SU transcription factors was found in the naive subtype – perhaps this implies a requirement for this number in maintaining a transcriptional programme poised for commitment to one of several possible differentiated fates. A reduced number of TFs may then be required to maintain a more-specialised fate after differentiation.”

Also, are there any examples of signature genes whose expression profiles do not match with what is assumed in the literature, or known ‘markers’ from the literature that fail to show up as signature genes? This would help to place into context the mentioned examples of signature genes that match what is known in the literature.

**Authors’ response:***In general, our findings agree well with previously characterized markers and we discuss occasions where our findings are not in complete concordance with previous work – this is particularly the case for the expression of some cytokines in resting cells (discussed on page 7). A valuable element of this work is the prediction of novel candidate markers that have not been described previously – we discuss this within the manuscript and hope that the online resource at**www.th-express.org**is valuable in further exploration of these genes.*

As a final remark, the authors are inferring the 'activities' of TFs in different subtypes solely from their mRNA expression levels but TFs often vary in activities do to other regulatory mechanisms (I already commented on this above). Such non-transcriptional changes in activity can be inferred from the expression behavior of the targets of the TFs. My group has actually developed a tool to automatically infer such TF activities from raw RNA-seq data (it is available at ismara.unibas.ch and only requires uploading the data). It might be interesting for the authors to compare the mRNA level changes of key TFs with the activity changes inferred from the behavior of the targets of these TFs.

**Authors’ response:***This would be an interesting use of these data but we feel that it is beyond the scope of the current study. We will certainly use this tool in future explorations of these data and we anticipate that our data will generally be valuable in the inference of TF activities as well as in the creation of regulatory networks.*

Finally, I could not follow the analysis of splice variation. The authors say that the major isoform comprises 80-90% of the total expression FOR EACH protein-coding gene. Are they really saying this is true for ALL genes? The legend of Figure 11 is not really specific about what is shown (this is actually something that can be improved in many of the legends) but my guess is that what it shows is that, for the genes shown, the interquartile range for the frequency of the major isoform is 80 = 90%. This would mean that the 50% of 'typical' genes have between 80-90% in the major isoform.

**Authors’ response:***We apologise for the lack of clarity here. Your interpretation is correct. We have altered the text in the results section as follows. We have also altered figure legends throughout the manuscript to add clarity.*

“For protein-coding genes with multiple isoforms we typically observed a major isoform that comprised the majority of the gene’s total expression while any further isoforms contributed decreasing proportions (Figure 11 A and B). This is similar to the distribution of isoforms previously described in human cells [51].

Quality of written English: Acceptable

## Additional file

Additional file 1:
**Subtype upregaulted genes for each CD4** ^**+**^ **subtype and lists of genes likely to encode transcription factors, cytokines and receptors.**

## Abbreviations

HE: Highly expressed
iTreg: Induced regulatory T cell
LE: Lowly expressed
MISO: Mixture of isoforms model
PSI: Percent spliced in
rlog: Regularized log
SU: Subtype upregulated
TF: Transcription factor
Treg: Regulatory T cell
